# Perfluoroalkyl Chain Length Effect on Crystal Packing and [LnO_8_] Coordination Geometry in Lanthanide-Lithium β-Diketonates: Luminescence and Single-Ion Magnet Behavior

**DOI:** 10.3390/ijms24119778

**Published:** 2023-06-05

**Authors:** Kristina A. Smirnova, Yulia O. Edilova, Mikhail A. Kiskin, Artem S. Bogomyakov, Yulia S. Kudyakova, Marina S. Valova, Galina V. Romanenko, Pavel A. Slepukhin, Victor I. Saloutin, Denis N. Bazhin

**Affiliations:** 1 International Tomography Center, Siberian Branch of the Russian Academy of Sciences, 630090 Novosibirsk, Russia; k.smirnova@tomo.nsc.ru (K.A.S.); bus@tomo.nsc.ru (A.S.B.); romanenk@tomo.nsc.ru (G.V.R.); 2 Postovsky Institute of Organic Synthesis, Ural Branch of the Russian Academy of Sciences, 620137 Yekaterinburg, Russia; edilova_yuliya@mail.ru (Y.O.E.); valova.ios.uran@mail.ru (M.S.V.); slepukhin@ios.uran.ru (P.A.S.); saloutin@ios.uran.ru (V.I.S.); 3 N.S. Kurnakov Institute of General and Inorganic Chemistry, Russian Academy of Sciences, 119991 Moscow, Russia; mkiskin@igic.ras.ru; 4 Department of Organic and Biomolecular Chemistry, Ural Federal University Named after the First President of Russia B.N. Eltsin, 620002 Ekaterinburg, Russia

**Keywords:** β-diketonates, lanthanide, luminescence, single-ion magnet, crystal packing

## Abstract

Functionalized perfluoroalkyl lithium β-diketonates (LiL) react with lanthanide(III) salts (Ln = Eu, Gd, Tb, Dy) in methanol to give heterobimetallic Ln-Li complexes of general formula [(LnL_3_)(LiL)(MeOH)]. The length of fluoroalkyl substituent in ligand was found to affect the crystal packing of complexes. Photoluminescent and magnetic properties of heterobimetallic β-diketonates in the solid state are reported. The effect of the geometry of the [LnO_8_] coordination environment of heterometallic β-diketonates on the luminescent properties (quantum yields, phosphorescence lifetimes for Eu, Tb, Dy complexes) and single-ion magnet behavior (*U*_eff_ for Dy complexes) is revealed.

## 1. Introduction

Advances in lanthanide coordination chemistry continues to be the cornerstone in the development of promising sensitizers, agents for theranostics, catalysts, and optical and magnetic materials [1,2,3,4,5,6,7,8]. Exploring the luminescent and magnetic properties of Ln^III^ compounds allows the structure–property correlations to be determined for further optimization of the ligand environment.

One of the bright areas of β-diketones application as ligands is the sensitization of Ln^III^ ions [9,10,11,12]. A number of lanthanide complexes (mainly Ln = Eu^III^ and Tb^III^) containing fluorinated β-diketonates were found to exhibit both long luminescence lifetimes and high photoluminescence quantum yields (PLQY) [13,14,15,16,17,18,19]. The main strategy to improve the luminescent properties of Ln^III^ complexes based on β-diketonates includes using the heterocyclic coligands with an extended π-system, phosphine oxides, sulfoxides [13,14,15,16,17,18,19,20,21,22]. Increasing the length of fluoroalkyl groups in β-diketonate anions is another important tool in fine-tuning the optical properties of compounds [17,18,23]. However, less attention has been paid to the ligand structure and cation nature variation when designing Ln^III^ *tetrakis*-diketonates [24,25,26,27,28,29,30,31]. Despite this, the directed synthesis of compounds with a [EuO_8_] environment is highly relevant, which is confirmed by the high quantum yield value (equal to 85%) reported for Eu(dik)_3_(Ph_2_SO)_2_ [32].

Over the past two decades, the magnetic properties of lanthanide complexes have been extensively studied. In particular, the introduction of the Dy^III^ ion into a suitable ligand environment remains among the most efficient strategies in the design of single-ion magnets (SIMs) for advanced memory storage technologies and spintronic devices [33,34,35]. The ligand structure manipulation has been regarded to increase the energy barrier for spin reversal and/or blocking the temperature of magnetization. While a considerable effort has been made to enhance the SIMs performance, more detailed approaches are still required to elucidate the origin of slow relaxation, as well as the impact of ligand structure and crystal packing to obtain the improved SIMs. According to previous studies on high-performance SIMs, highly symmetrical Dy^III^-based SIMs, such as D_4d_, D_5h_ and D_6h_, provide reduced electron repulsion around the lanthanide ion and the ^m^J = ±15/2 state stabilization, thereby preventing the quantum tunnelling magnetization (QTM) [36,37,38,39,40]. In this context, the structures having approximately square-antiprismatic or dodecahedral coordination polyhedra are of much interest because of their high potential as SIMs. The magnetic relaxation behavior of these SIMs has been shown to be affected by distortion of the coordination sphere, typically caused by subtle modification of the lattice solvent, auxiliary ligand, and intermolecular interaction [41,42,43,44,45,46,47,48]. Therefore, the anisotropy of the lanthanide ions and their magnetic dynamics are strongly influenced by both the ligand field and coordination geometry [36,37,38,39,40,41,42,43,44,45,46,47,48,49,50,51]. However, it is still a big challenge to control the coordination geometry around the lanthanide ions in order to understand how it affects the relaxation mechanism. For this reason, shaping the geometries with different organic ligands has become the main strategy in most studies.

One of the first examples of a single-molecular magnet was *tris*-diketonate [Dy(acac)_3_·2H_2_O], in which the replacement of water molecules by diazaaromatic ligands increased the energy barrier [52,53,54]. Since then, β-diketonates have remained an essential tool in the field of molecular magnetism [55,56,57,58,59,60,61,62,63,64,65]. Varying different substituents in the diketonates can modify the lanthanide crystal field, including by changing the geometry of [LnO_8_] or [LnO_7_] polyhedra [51,61,64]. Furthermore, the electrostatic potential (ESP) around the central Dy^III^ ions indirectly depends on the electron-donating/withdrawing properties of the auxiliary ligands. This opens the way to the regulation of magnetic dynamics, especially in the suppression of fast relaxation [62]. In particular, the electron-withdrawing effect of fluorinated β-diketone was demonstrated to increase the magnetic relaxation barriers [62].

However, in heterometallic lanthanide-alkali metal β-diketonates, the Ln^III^ ion is surrounded by four identical β-diketones to form the [LnO_8_] environment [64,65,66]. In this case, we can observe how minor changes in geometry of the coordination polyhedron [LnO_8_] induced by the different fluoroalkyl substituents affect the properties of the complexes. Therefore, this class of molecules provides an ideal model system for studying the crystal field effect on magnetic properties.

Because there are very few examples of such compounds, their detailed magnetic studies are scarce to date. As far as we know, slow magnetization relaxation has been reported for only two complexes of this type: [Cs{Dy(Ph_2_acac)_4_}]_n_ (Ph_2_acac = dibenzoylmethanide) and [M{Ln(hfac)_4_}] (M = K, Cs; Ln = Dy, Er) [64,65].

**Figure 1:**
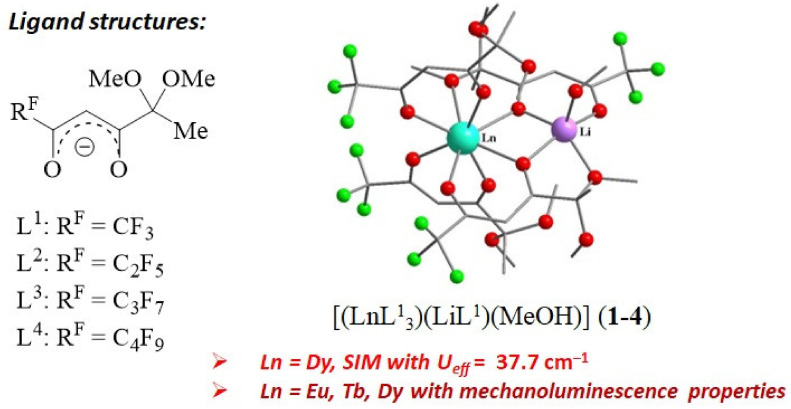
Structures of β-diketonates used in this study [66,68].

Previously, we have synthesized a series of heterometallic complexes based on asymmetric fluorinated β-diketonates (L^1^, L^2^) (Figure 1) [66,67,68,69,70,71,72]. To the best of our knowledge, these structures are the first described examples of [Ln-Li] β-diketonates. Their luminescent and magnetic properties have been studied in detail [66]. Particularly, the single-molecular magnet properties were found for [(DyL^1^_3_)(LiL^1^)(MeOH)] (**1**) with trifluoromethylated β-diketonate as **L^1^** (Figure 1). Its energy barrier separating two opposite magnetic states was 37.7 cm^−1^ [66].

The current work aims to investigate the correlations between the structure and magneto-optical properties of Ln^III^ complexes with a similar [LnO_8_] coordination environment. By varying the fluoroalkyl substituent in the β-diketonates (from L^1^ (R^F^ = CF_3_) to L^4^ (R^F^ = C_4_F_9_)), we have demonstrated how the crystal packing, intermolecular interactions, and minor changes of [LnO_8_] geometry influence the properties of luminescent and magnetoactive molecules. 

## 2. Results and Discussion

### 2.1. Synthesis of Dinuclear [Dy-Li] β-Diketonates

**Scheme 1:**
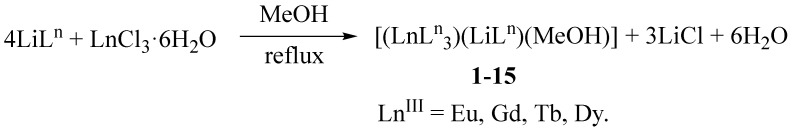
Synthesis of heterometallic complexes **1**–**15**.

**Scheme 2:**
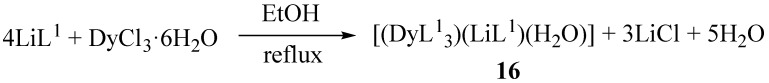
Synthesis of heterometallic complex **16**.

Ln^III^-Li^I^ diketonates **1**–**16** were synthesized through the reaction of functionalized fluorinated diketonates with corresponding lanthanide(III) chloride in methanol or ethanol as a solvent (Scheme 1 and Scheme 2), Table 1) according to the previous reports [66,68,70]. Changing the solvent from methanol to ethanol leads to the formation of heterometallic diketonate **16**, in which the geometry of the [LnO_8_] coordination polyhedron and crystal packing differ from those of complex **4** [66].

### 2.2. Structure of Dinuclear [Ln-Li] β-Diketonates

The structures of CF_3_-containing [Ln-Li] β-diketonates **1**–**4**, **7** and **16** have been described before [66,68,70]. In this work, we have synthesized and characterized heterometallic complexes **5**, **6**, **8**–**15**.

**Figure 2:**
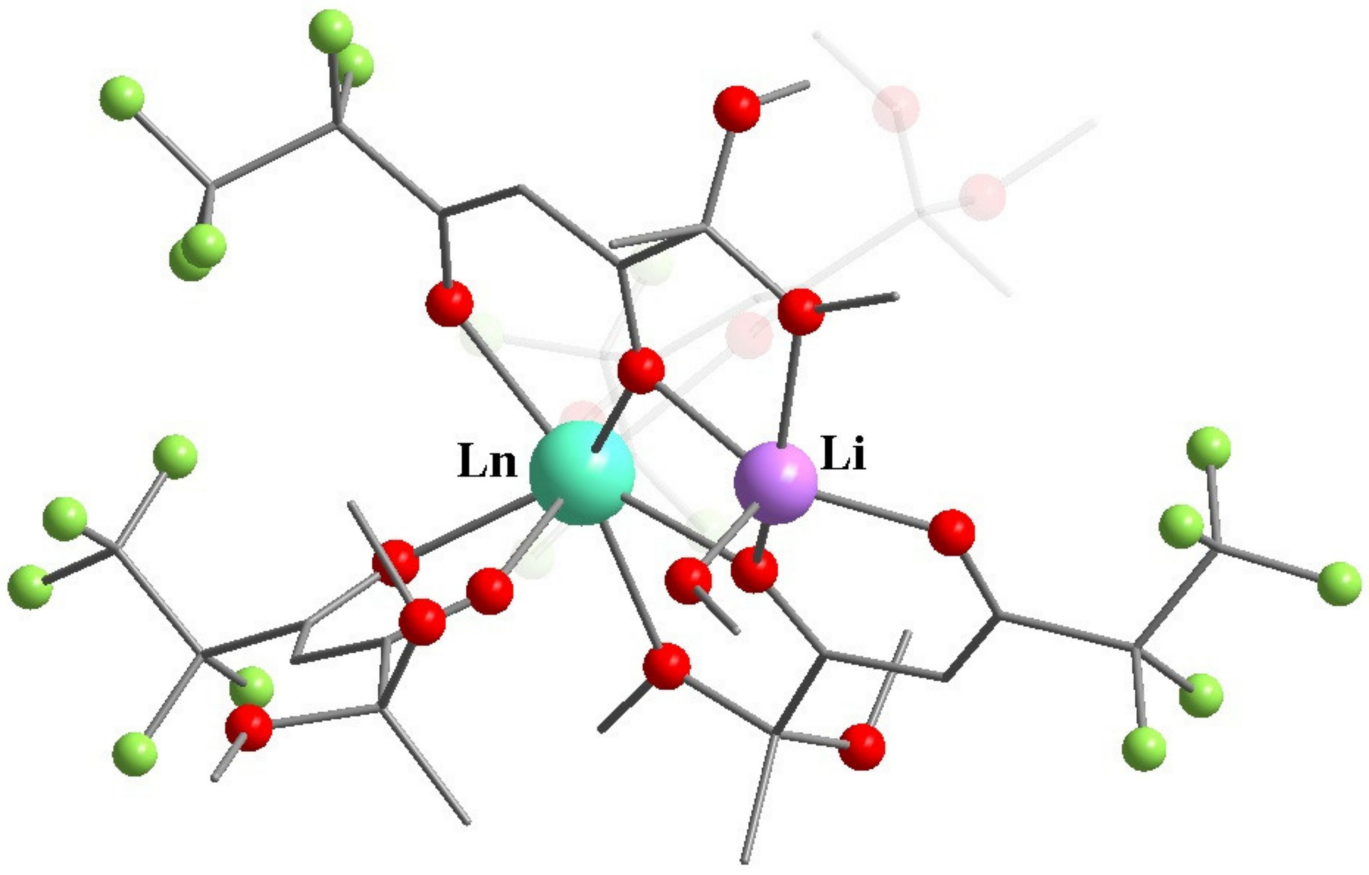
Molecular structure of heterometallic complexes **5**–**8** (dik = L^2^, Ln = Eu, Gd, Tb, Dy). Hydrogen atoms are omitted and one diketonate ligand is transparent for clarity.

**Figure 3:**
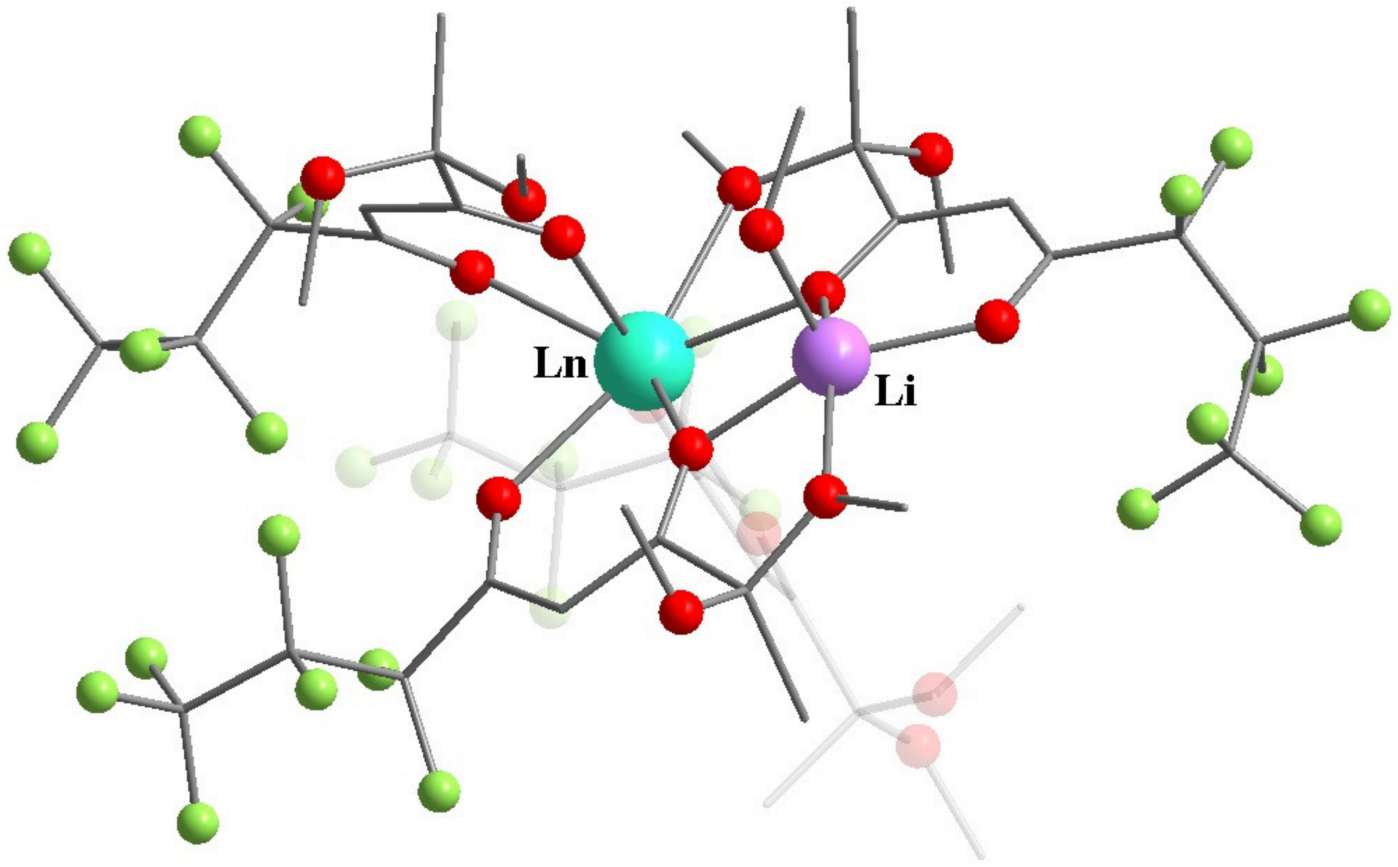
Molecular structure of heterometallic complexes **9**–**11** (dik = L^3^, Ln = Eu, Gd, Tb). Hydrogen atoms are omitted and one diketonate ligand is transparent for clarity.

**Figure 4:**
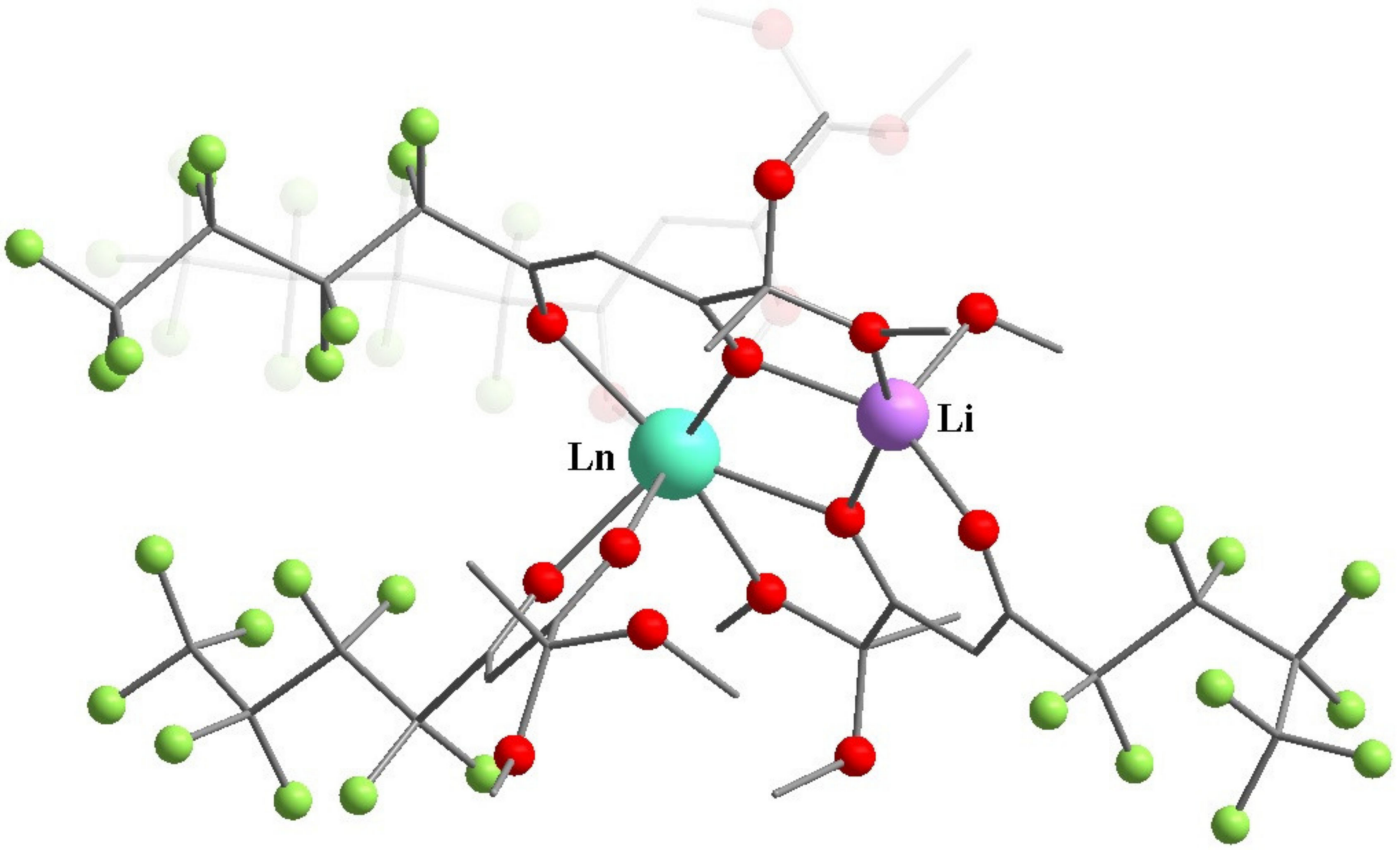
Molecular structure of heterometallic complexes **12**–**15** (dik = L^4^, Ln = Eu, Gd, Tb, Dy). Hydrogen atoms are omitted and one diketonate ligand is transparent for clarity.

Based on the XRD data, the structures of novel discrete heterometallic complexes **5, 6, 8**–**15** are similar to that of trifluoromethylated analogue **1** and correspond to a composition of [(LnL_3_)(LiL)(MeOH)] (Figure 2, Figure 3 and Figure 4, Table 1 and Table S1). XRD powder patterns of complexes **4**, **7**, **10**, **14** with different **L** coincide with the theoretical ones calculated for the dysprosium(III), terbium(III) and gadolinium(III) complexes (Figures S2–S5).

**Figure 5:**
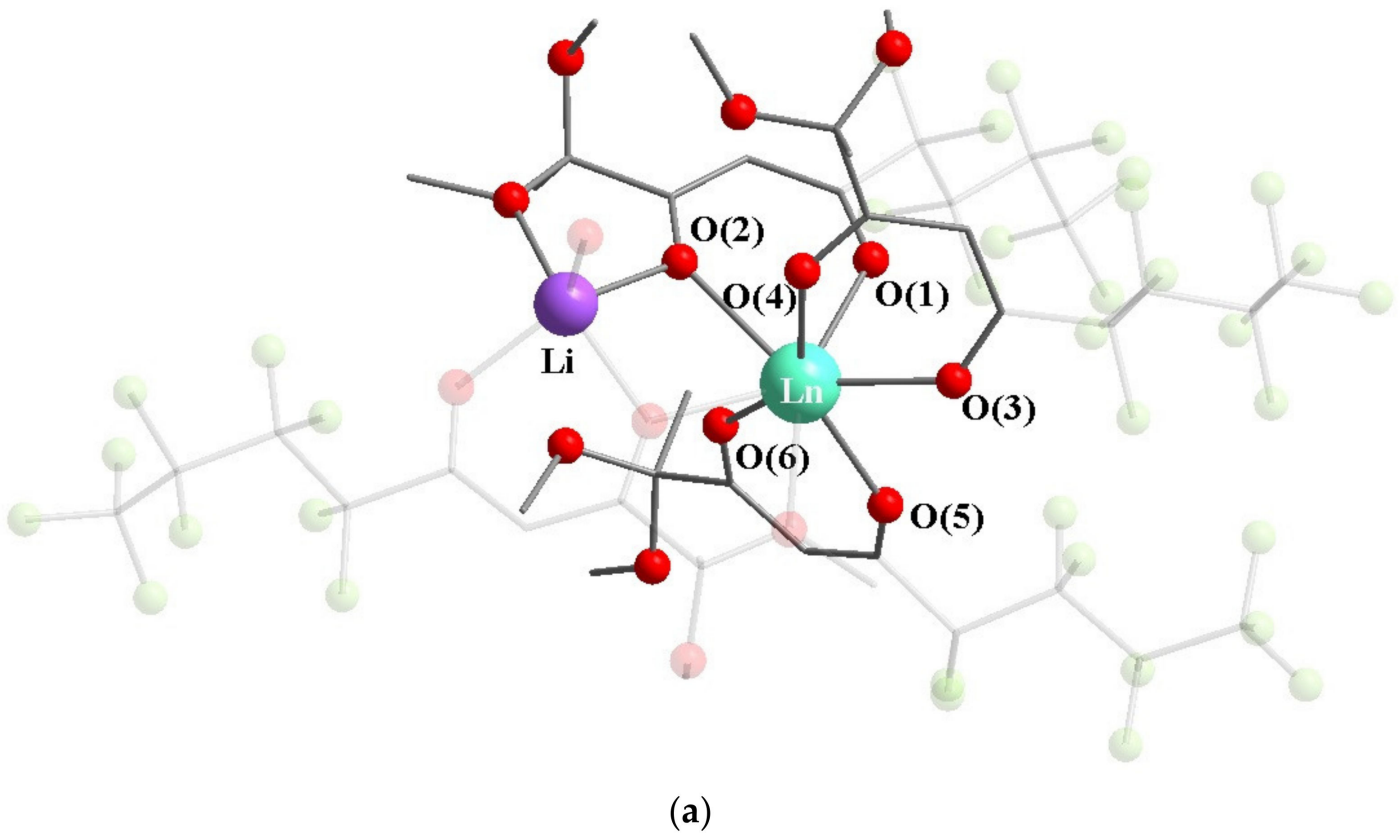

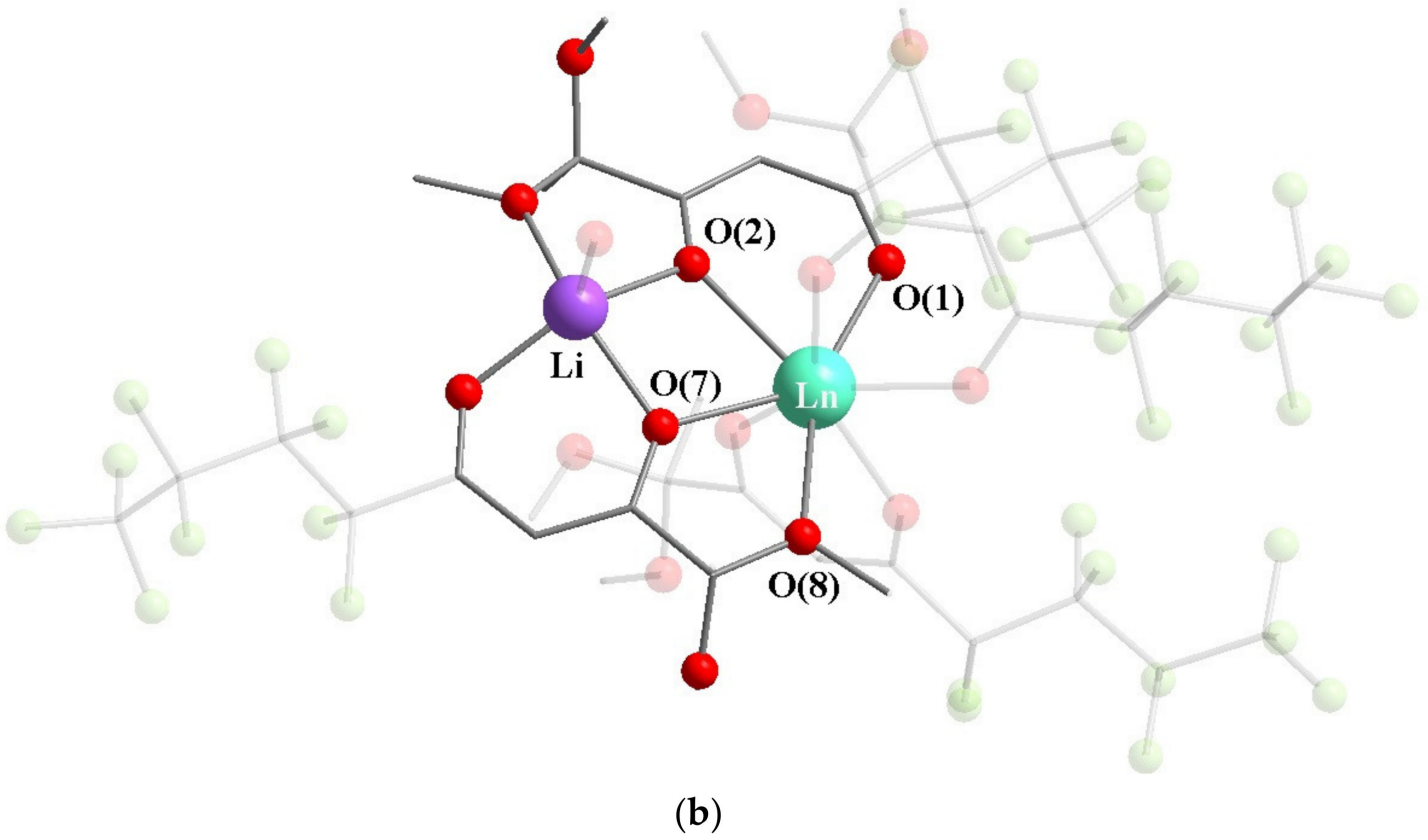
The structures of Ln *tris*-diketonate (**a**) and heterometallic framework (**b**) shown for L^4^-based complexes.

In the series of complexes **1**–**16**, the coordination sphere of Ln atoms includes eight oxygen atoms O(1) → O(8) from four β-diketonate molecules. Three ligand molecules form three six-membered 4f-metallocycles due to the β-diketonate-anions and result in a *tris*-diketonate lanthanide fragment (Figure 5a). The fourth β-diketonate molecule is an initial LiL, which coordinates the lanthanide ion via one methoxy group (O(7)) and oxygen atom of the β-diketonate-anion (O(8)) to give a heterometallic complex structure (Figure 5b). Similarly, one of the molecules from the *tris*-diketonate fragment coordinates with the Li atom. Methanol molecule completes the coordination sphere of penta-coordinated Li(I) ion. The values of the O-Ln-O angles in the five- and six-membered metallocycles of complexes **1**–**16** and the bond lengths between the Ln and O(1)–O(8) atoms are given in SI (Tables S2–S9).

In complexes **1**–**16**, the bond lengths of the Ln atom with the bridging oxygen atoms O(2) and O(8) are reasonably longer than the corresponding Ln-O bonds of the *tris*-diketonate fragment (Tables S6–S9). Varying the length of the fluoroalkyl substituent from the CF_3_ to C_3_F_7_ group in diketonates leads to obvious changes in both the Li···Ln distance (~3.49 vs. 3.53) and the Li-O(7)-Ln angle (~104° vs. 106°) (Tables S2–S9).

The shortest distances between the Ln centers in complexes indicate some specific features of their crystal packing (Figures S6–S17). For example, the Ln···Ln distance increases only when the L^1^ diketonate is replaced by L^2^ one. Trifluoromethyl complexes [LnL^1^_3_)(LiL^1^)(MeOH)] **1**–**4** with the shortest Ln···Ln distance form a zigzag chain with ∠Ln(1)-Ln(2)-Ln(3)~131° (Figures S3–S5). However, in the case of L^2^-L^4^ diketonates, we observe linear 1D chains, which lead to layered crystal structures of complexes (Figures S7–S9). The shortest distance between the two adjacent molecules of complex **16** is determined by the presence of intermolecular hydrogen bonds of the coordinated water without molecule chain motif.

Using the SHAPE program [73,74], we have calculated the geometry of coordination [LnO_8_] polyhedra of synthesized heterometallic β-diketonates **1**–**16** (Table 2, also see Tables S10–S13). Table 2 shows that increasing the length of fluoroalkyl group in diketonates strongly distorts the triangular dodecahedron (TDD-8) geometry of [LnO_8_] polyhedron in Eu and Gd complexes. Calculated values of coordination polyhedra of Tb and Dy complexes are closer to those of the ideal TDD-8 geometry, except for C_3_F_7_ containing diketonate. In compound **16**, the calculated values of the distorted geometry of the Dy coordination environment correspond better to TDD-8 and biaugmented trigonal prism (BTPR-8).

To gain more insight into the solid structure analysis of obtained complexes, we have compared the dihedral angles between the LnOO and β-diketonate planes (Table S14). The length of the fluoroalkyl group in diketonates greatly affects the planarity of the five- and six-membered chelate cycles. As follows from Table S14, the sum of the dihedral angles between the LnOO and β-diketonate planes increases from CF_3_ (L^1^) to C_4_F_9_ (L^4^) complexes. The exception is C_2_F_5_ β-diketonates (L^2^), for which the deviations from the planarity of the chelate cycles are the smallest in this series. For the five-membered chelate cycles of the complexes, the values of dihedral angles vary irregularly with increasing fluorine atoms in β-diketonates (Table S14).

The IR spectra of **1**–**16** are similar and display strong absorption bands in the range of 1645–1633 and 1541–1468 cm^−1^, which is typical for β-diketonates, that can be attributed to enolate C=O and C=C vibrations, respectively (Figure S18) [16]. Typical C–F vibrations are observed in the range of 1198–1122 cm^−1^ (the most intensive peaks are at 1137–1122 cm^−1^), and C–H vibrations can be seen between 3000 and 2840 cm^−1^. 

### 2.3. Mechanoluminescence

Previously, we have observed the mechanoluminescence (ML) of polycrystalline trifluoromethyl Ln-Li diketonates **1**, **3** and **4** [66]. However, complexes based on ligands with R^F^ ≥ C_2_F_5_ are ML-inactive **5**, **7**–**9**, **11**, **12**, **14**, and **15**. This indicates the crucial role of structural differences in the crystal packing for ML activity of the complexes.

The crystal packing of trifluoromethyl diketonates **1**, **3**, **4** consists of zigzag 1D chains of isolated molecules of complexes with the shortest distance between Ln atoms (Figures S7–S9). In complexes based on ligands with R^F^ ≥ C_2_F_5_, molecules are arranged lengthwise to form linear 1D chains. The coordinated water molecule in complex **16** forms hydrogen bonds to give dimers as the structural unit with the shortest Dy···Dy distance (Figure S17). Therefore, the molecular herringbone arrangement is a feature of ML activity that is consistent with the reported examples of Ln^III^ *tris*-diketonates [13].

### 2.4. Photoluminescence

**Figure 6:**
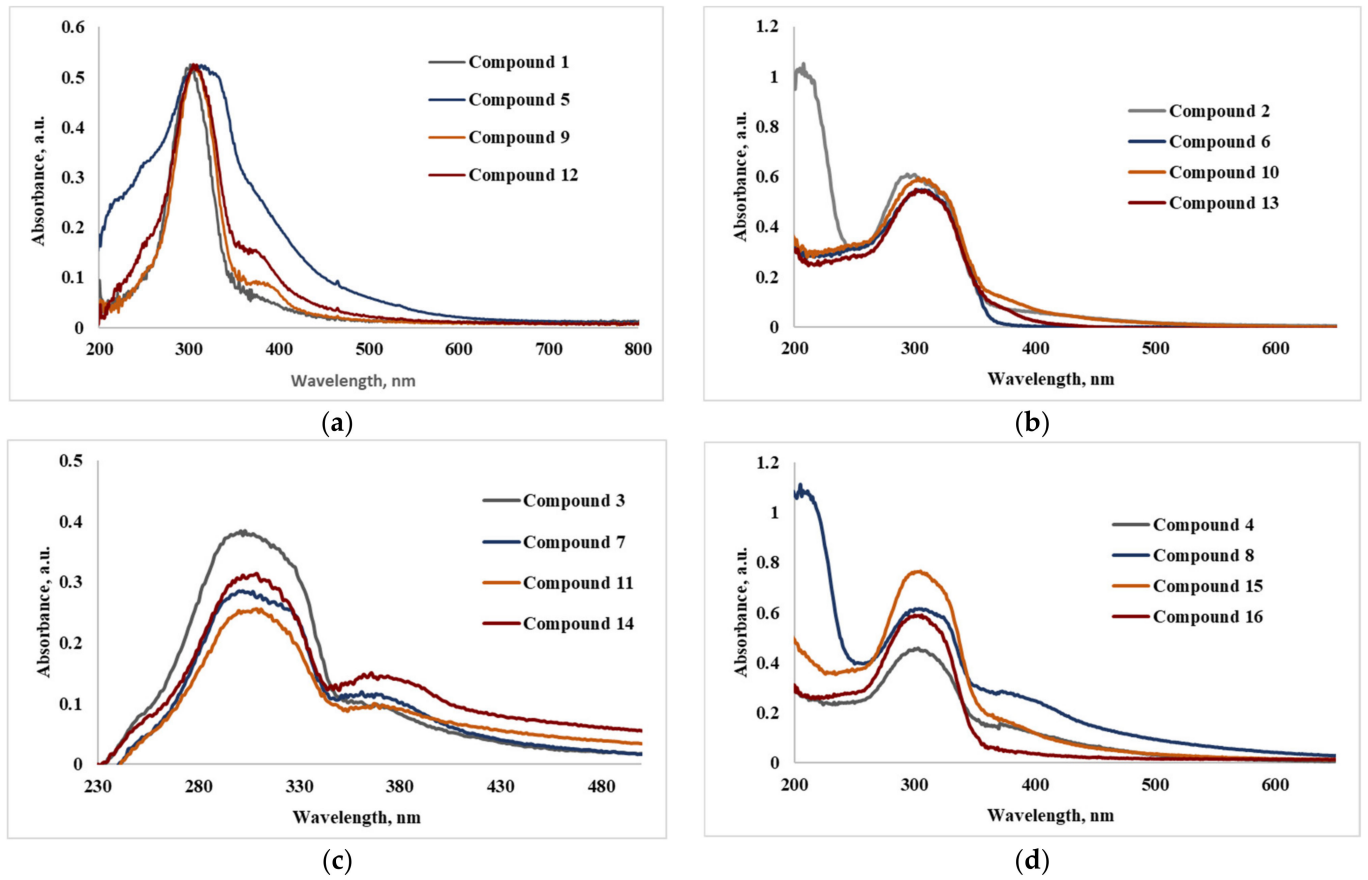
UV-VIS spectra of Eu^III^ complexes **1**, **5**, **9** and **12** (**a**), Gd^III^ complexes **2**, **6**, **10** and **12** (**b**), Tb^III^ complexes **3**, **7**, **11** and **14** (**c**), Dy^III^ complexes **4**, **8**, **15** and **16** (**d**).

**Figure 7:**
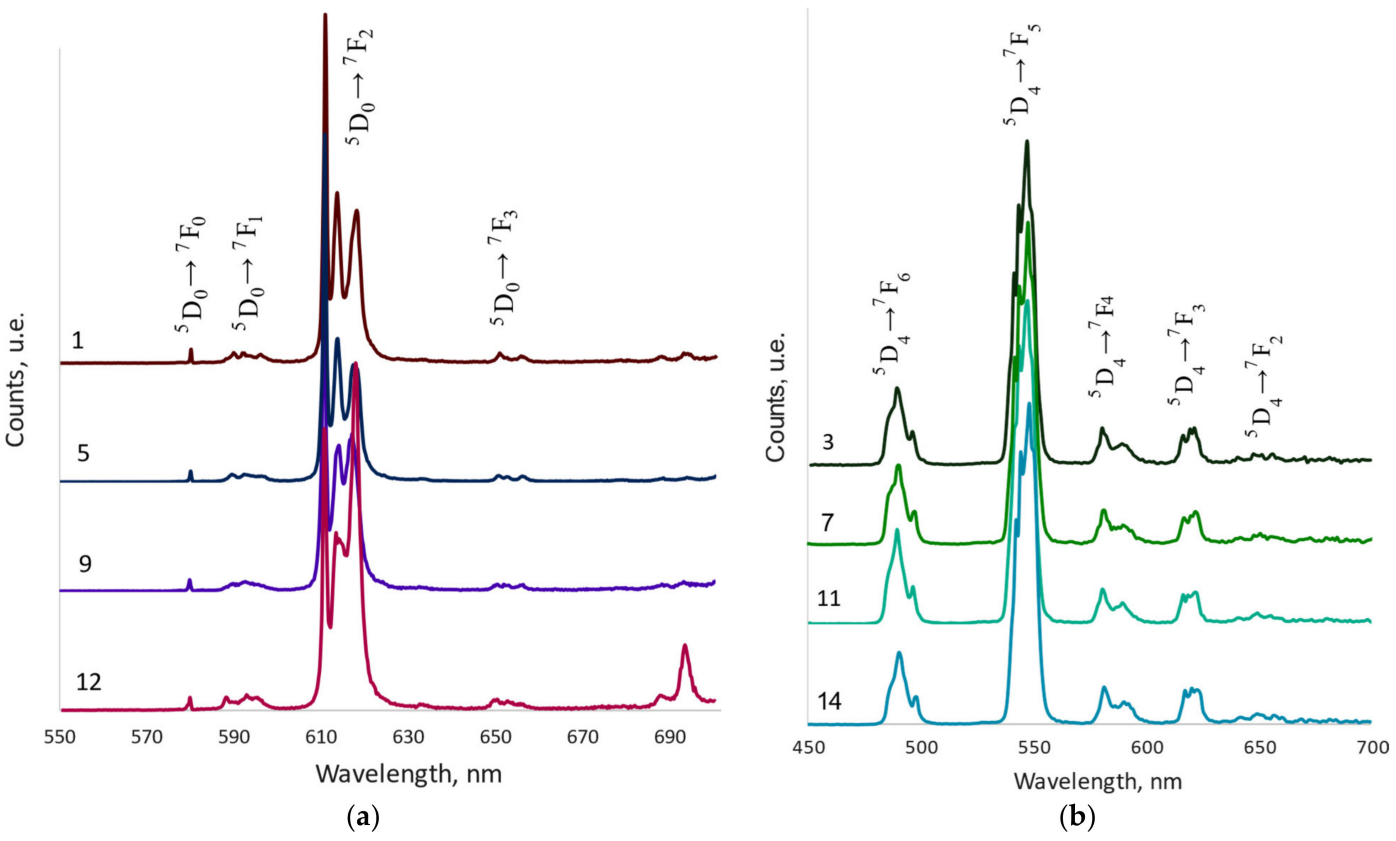
Photoluminescence spectra of Eu^III^ complexes **1**, **5**, **9** and **12** (**a**) and Tb^III^ complexes **3**, **7**, **11** and **14** (**b**).

**Figure 8:**
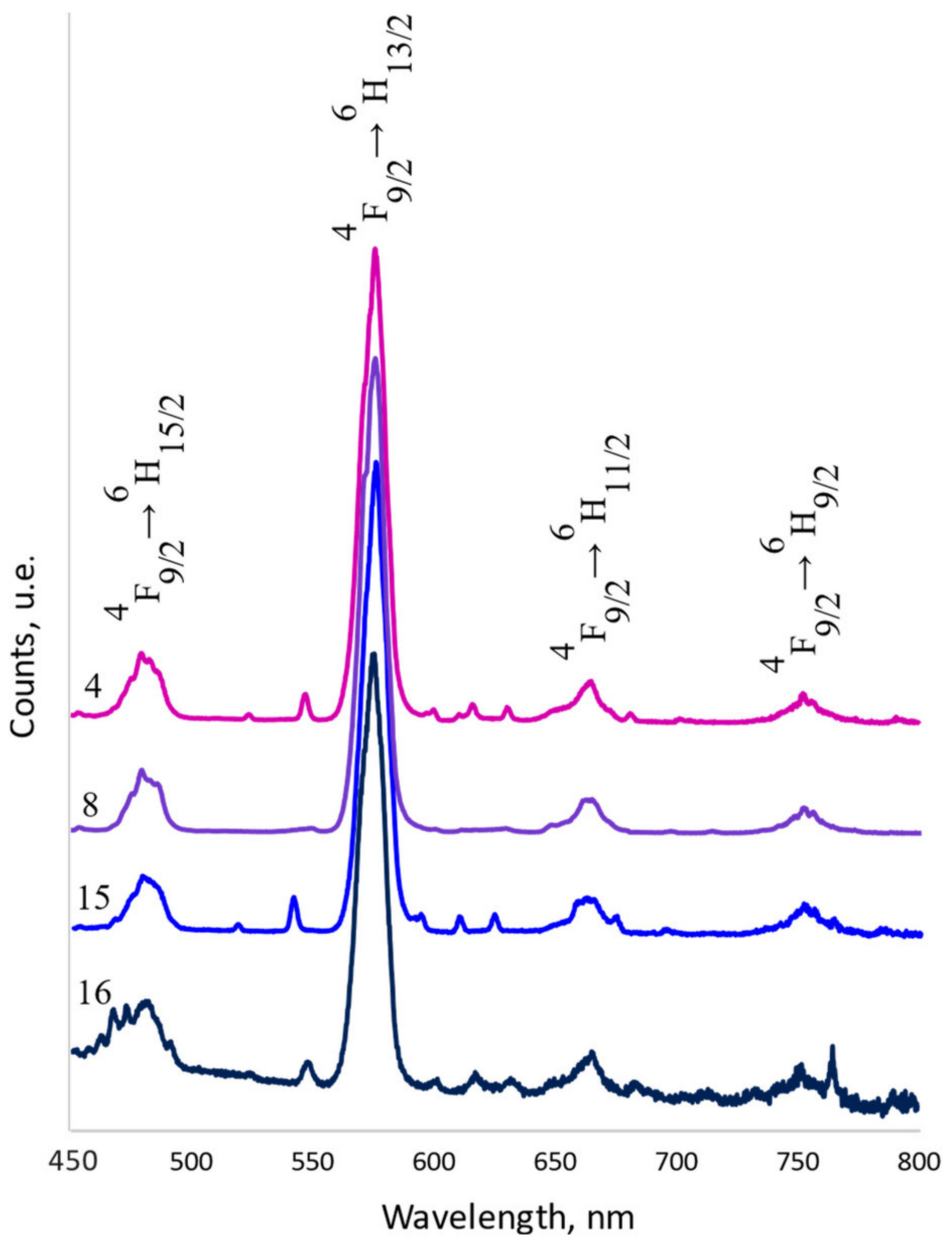
Photoluminescence spectra of Dy^III^ complexes **4**, **8**, **15** and **16**.

The photoluminescence properties of Eu^3+^, Tb^3+^ and Dy^3^+ complexes are studied in the solid state. Complexes under illumination by a standard laboratory UV lamp at 365 nm clearly showed visible red, green and yellow luminescence depending on the nature of the lanthanide ion. Broad excitation band in the UV region of the spectrum with the maximum at 320–330 nm indicates the ligand-centered absorbance resulted from π* ← π absorption of β-diketonate fragment L (Figure 6). Upon excitation at 340 nm, complexes showed the characteristic emission bands of the corresponding Ln^III^ ion originating from the following transitions: ^5^D_0_ → ^7^F_J_ for **1**, **5**, **9**, **12** (Figure 7a); ^5^D_4_→ ^7^F_J_ for **3**, **7**, **11**, **14** (Figure 7b); and ^4^F_9/2_ → ^6^H_J_ for **4**, **8**, **15**, **16** (Figure 8).

We have calculated the radiative ($A_{rad}$) and non-radiative ($A_{nrad}$) decay rates, as well as the ^5^D_0_ intrinsic quantum yield ($Q_{Ln}^{Ln}$) of the complexes using the following Equations:

$$A_{rad}=A_{MD,0}n^{3}\frac{I_{total}}{I_{MD}}\;A_{nrad}=\frac{1}{\tau^{obs}}-A_{rad}\;Q_{Ln}^{Ln}=\tau^{obs}\cdot A_{rad}$$

where A_*MD*,0_ is the spontaneous emission probability of the magnetic dipole ^5^D_0_ → ^7^F_1_ transition, which is equal to 14.65 s^−1^; *n* is the refractive index (considered to be 1.5 for solids); (I*_total_*/I*_MD_*) is the ratio of the total integrated area of the corrected Eu^3+^ emission spectrum to the area of the ^5^D_0_ → ^7^F_1_ band [13,75]; and $\tau^{obs}$ is measured ^5^D_0_ luminescence lifetime of [EuL_3_)(LiL)(MeOH)] **1**, **5**, **9**, **12** (Table 3, Figures S24, S27, S30 and S32). 

Sensitization efficiency (${ŋ}_{sens}$) for the Eu^III^ complex was estimated as the ratio of the measured ^5^D_0_ luminescence quantum yield ($Q_{Ln}^{L}$) to the calculated intrinsic quantum yield (Table 3):

$${ŋ}_{sens}=Q_{Ln}^{L}\;/Q_{Ln}^{Ln}$$

To elucidate the energy transfer process of the lanthanide(III) complexes, the triplet energy levels of the ligands were estimated. Since the lowest lying excited level (^6^P_7/2_ → ^8^S_7/2_) of Gd^3+^ is located at 32,150 cm^−1^, we can determine the ^3^ππ* energy levels of ligand L^1^-L^4^ anions based on the phosphorescence spectra of [GdL_3_)(LiL)(MeOH)] (Figures S20–S23). The phosphorescence spectra of compounds **2** (L^1^), **6** (L^2^), **10** (L^3^) and **13** (L^4^) were recorded at 77 K and their zero-phonon transition energies of triplet states (3ππ*) corresponded to 443 nm (22,540 cm^−1^), 446 nm (22,420 cm^−1^), 445 nm (22,480 cm^−1^) and 450 nm (22,200 cm^−1^), respectively. The obtained values of L^1^-L^4^ triplet levels are lower than that of acac (24,800 cm^−1^) [62], but close to those of trifluoromethyl non-functionalized diketones, e.g., tfac (22,700 cm^−1^) and hfac (22,200 cm^−1^) [62].

Because of the large difference of energy levels in Eu^III^ complexes, the energy transfer from E_0-0_(3ππ*) to ^5^D_2_ is the most probable process for them [75]. In Tb^III^ complexes, the energy levels E_0-0_(3ππ*) and ^5^D_4_ are close, thus quenching of the luminescence intensity is possible due to the reverse energy transfer from the excited level of Tb^III^ to ligand.

Since the differences in the triplet levels of the ligands are insignificant, we consider the geometry of the Ln coordination environment as the key factor influencing the luminescence properties. It was previously reported that a low-symmetry ligand environment around Ln^3+^ ion (from *D*_4d_, *D*_2d_ to *C*_3v_, *C*_2v_) improves the luminescence efficiency [15]. This correlation is observed in the case of Eu^III^ and Dy^III^ complexes. In C_2_F_5_-diketonate-based complexes, the geometry of the coordination environment [EuO_8_] is closest to that of TDD-8 (*D*_4d_), which explains the difference of luminescence characteristics in this series of complexes. The largest deviation of the coordination polyhedron [DyO_8_] from the ideal TDD-8 geometry in the case of CF_3_-diketonate leads to a significant increase in the quantum yield. In contrast, a number of diketonates with C_2_F_5_/C_4_F_9_ substituents have a high quantum yield due to the [TbO_8_] geometry close to *C*_2v_ symmetry.

### 2.5. Magnetic Properties

**Figure 9:**
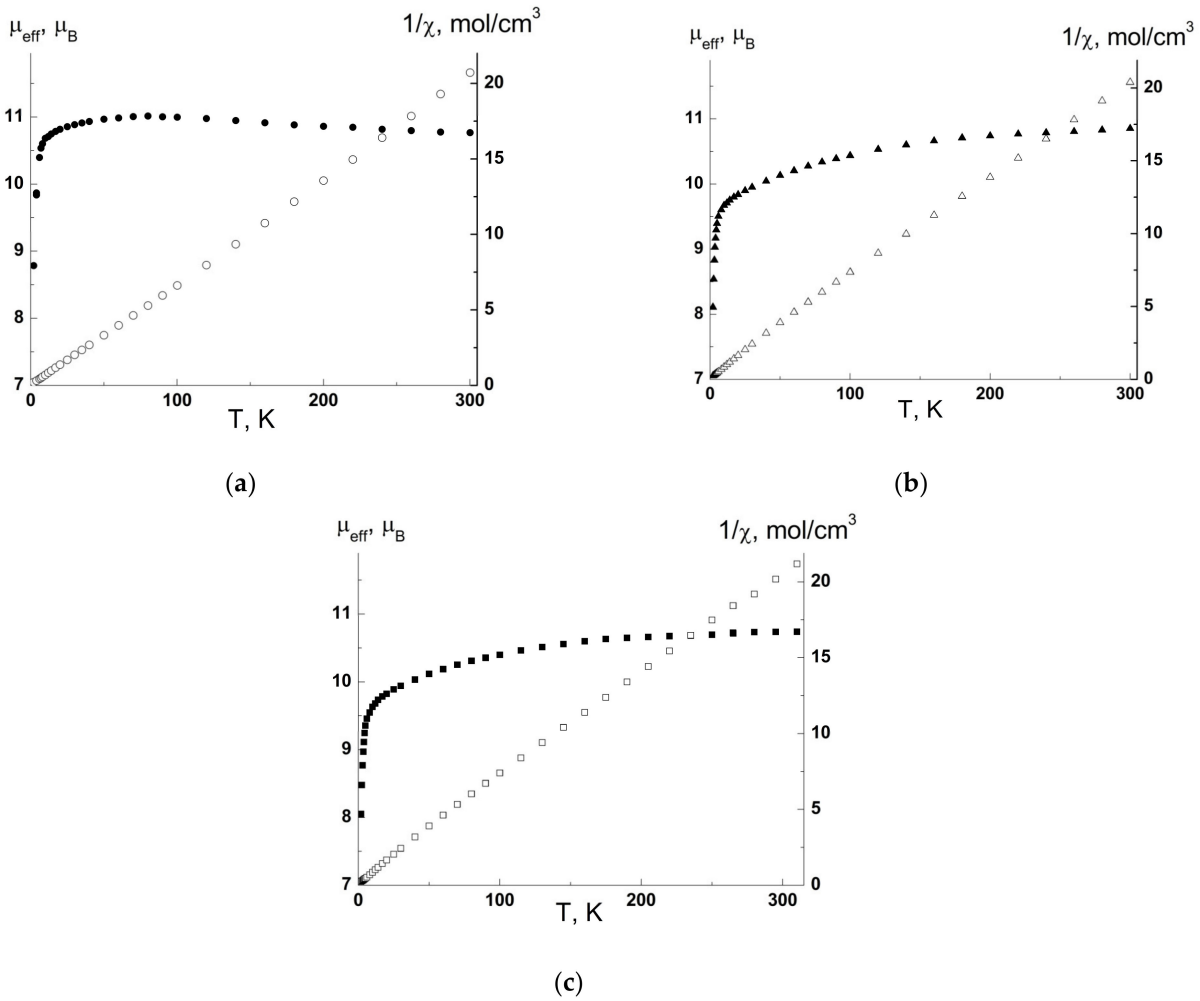
The *μ*_eff_(*T*) (filled points) and 1/χ(*T*) (empty points) dependences for complexes **8** (**a**), **15** (**b**) and **16** (**c**).

The effective magnetic moment *μ*_eff_ temperature dependences for polycrystalline samples of complexes **8**, **15**, **16** are shown in Figure 9. The room temperature values of *μ*_eff_ are 10.74–10.85 *μ_B_*, in accordance with the theoretical value of 10.64 *μ_B_* for Dy^III^ free ion (^6^H_15/2_ ground state with *g*_J_ = 4/3). Under cooling, *μ_eff_* gradually decreases for **15**, **16** and increases for **8** to 10 K below, and the moment drops to a value of 8–8.9 *μ_B_* by 2 K. Field dependencies of magnetization are nonlinear at low temperatures (Figures S38 and S39). The decrease of *μ*_eff_ at low temperatures suggests the mixture of anisotropy and possible intermolecular exchange interactions.

**Figure 10:**
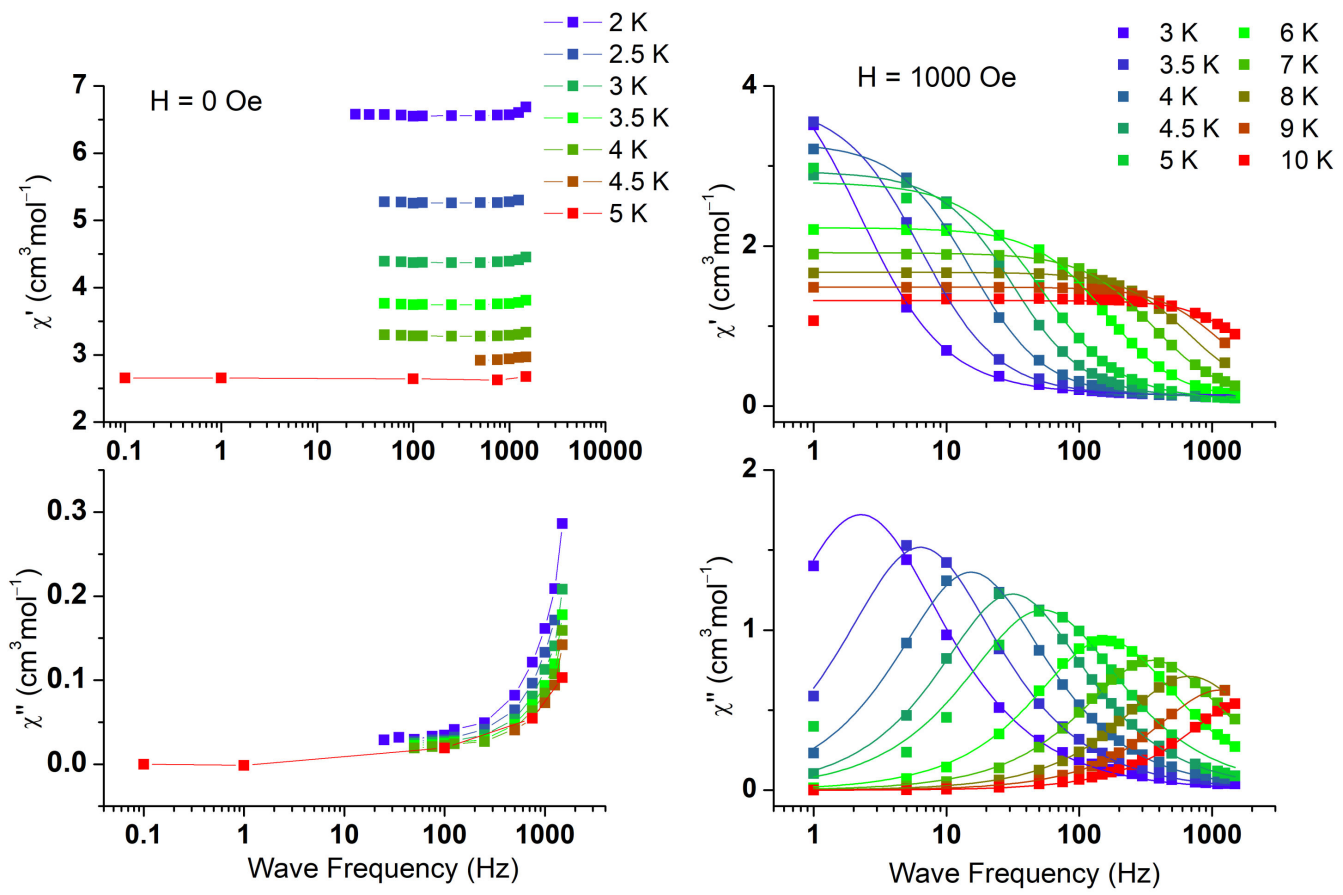
Frequency dependence of the in-phase (χ′) and out-of-phase (χ″) AC magnetic susceptibility for compound **8** under zero ((**left**), points are connected for clarity) and 1000 Oe (**right**) DC field; solid lines are fits to the Debye model.

AC magnetic measurements were performed for Dy^III^ complexes **8**, **15** and **16** (Figure 10, Figures S36 and S37). Fast magnetic relaxation is observed under zero dc field due to quantum tunneling magnetization. Applying dc field of 1000 Oe quenches QTM processes, and complexes **8**, **15** and **16** exhibit slow relaxation of the magnetization. Frequency dependences of the in-phase (χ′) and out-of-phase (χ″) AC magnetic susceptibility were analyzed using the generalized Debye model:

$$\chi^{\prime }\left(\omega \right)=\chi_{S}+\left(\chi_{T}-\chi_{S}\right)\frac{1+{\left(\omega \tau \right)}^{1-\alpha }\sin\left(\frac{\pi \alpha }{2}\right)}{1+2{\left(\omega \tau \right)}^{1-\alpha }\sin\left(\frac{\pi \alpha }{2}\right)+{\left(\omega \tau \right)}^{2-2\alpha }},\;\chi^{\prime \prime }\left(\omega \right)=\chi_{T}-\;\chi_{S}\frac{{\left(\omega \tau \right)}^{1-\alpha }\cos\left(\frac{\pi \alpha }{2}\right)}{1+2{\left(\omega \tau \right)}^{1-\alpha }\sin\left(\frac{\pi \alpha }{2}\right)+{\left(\omega \tau \right)}^{2-2\alpha }},\;$$

where χ_T_—adiabatic susceptibility, χ_S_—isothermal susceptibility, τ—relaxation time and α—relaxation times distribution width parameter. The best fit values of χ_T_, χ_S_, τ and α are listed in SI (Tables S15–S17).

**Figure 11:**
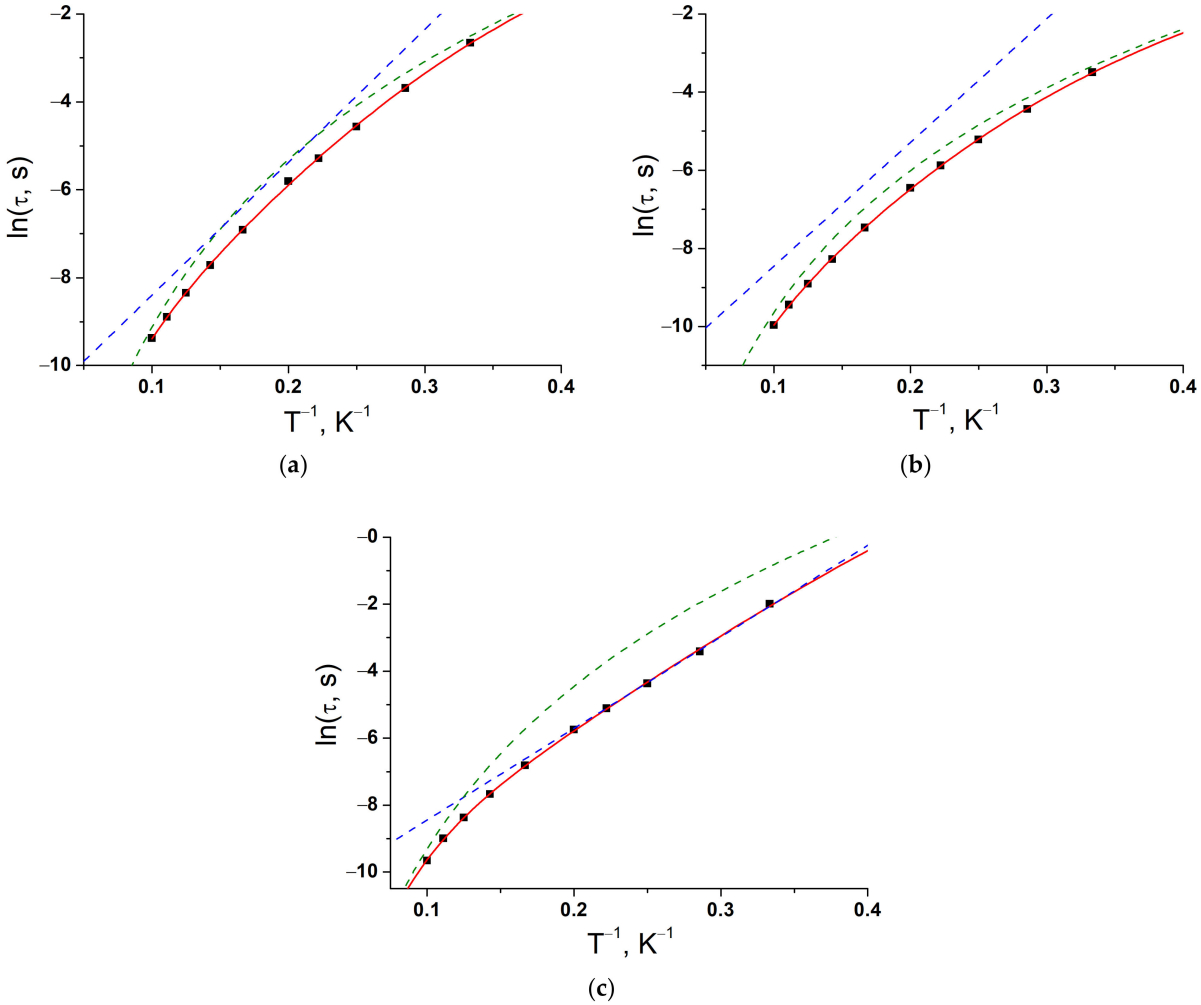
Arrhenius plots of relaxation time for complexes **8** (**a**), **15** (**b**), **16** (**c**) under 1000 Oe DC field. Solid lines are theoretical curves. Solid red lines are best-fitted curves for both Orbach and Raman processes, dashed blue and green lines are simulated curves for individual Orbach and Raman processes, respectively.

Arrhenius plots of relaxation time for complexes **8**, **15** and **16** are nonlinear (Figure 11), which implies more than just the Orbach relaxation mechanism ($\tau^{-1}=\tau_{0}^{-1}e^{-U_{\mathrm{eff}}/k_{B}T}$). Raman ($\tau^{-1}=CT^{n}$) and Direct ($\tau^{-1}=BH^{2}T$) processes are possible as alternative relaxation mechanisms, and the best fit of the experimental data for **8**, **15** and **16** was achieved by taking into account Orbach and Raman relaxation mechanisms [34,35,37]. Optimal parameter values *τ*_0_ and *U_eff_* for Orbach and *C* and *n* for Raman processes are listed in Table 4. Dashed lines in Figure 11 are simulated curves with the obtained best fit parameters for individual Orbach and Raman processes. Fitting the linear region of ln(τ) vs. *T* dependencies, considering only the Orbach relaxation mechanism, allows us to estimate energy barrier values *U*_eff_, which are close to those listed in Table 4. The obtained *U*_eff_ values for **8**, **15** and **16** are significantly lower than those for **4** (37.7 cm^−1^). In the case of **16**, the strong linear dependence of ln(τ) at a low temperature implies a domination of the Orbach relaxation mechanism. However, its curvature at a higher temperature indicates the competing relaxation processes, which makes it difficult to obtain Raman process parameters properly. Therefore, *n* = 7 was fixed to avoid overparameterization for **16**. The obtained values of *n* are expected for Kramer Ln^III^ ions, for which *n* may be in the range of 5–9; the deviation may be caused by differences in the crystal field strength [76]. 

**Figure 12:**
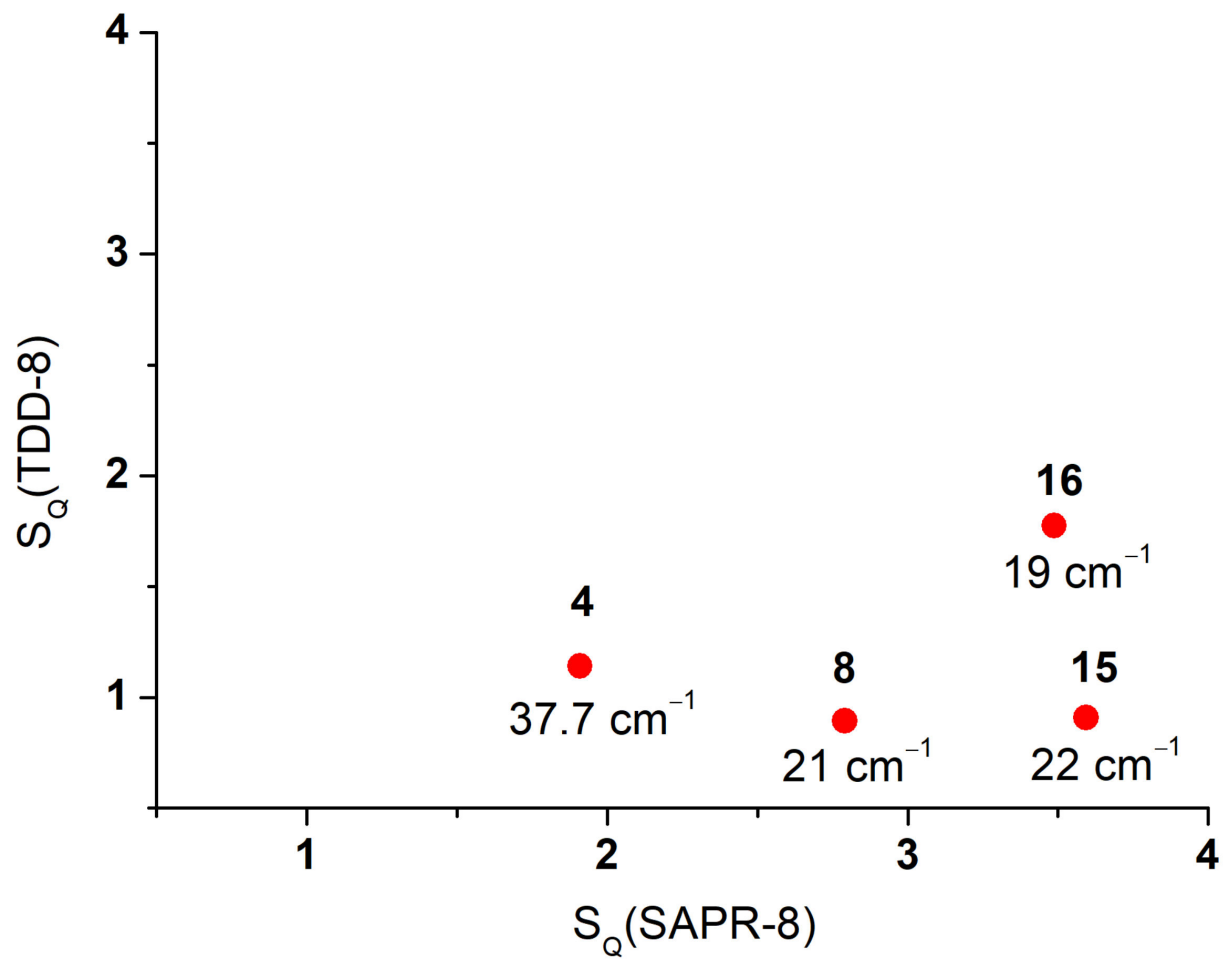
Continuous shape measures map (SAPR-8, square antiprism; TDD-8, triangular dodecahedron) for **4**, **8**, **15** and **16**. Red data points that correspond to each complex are additionally labeled with their U_eff_ values.

Magnetic anisotropy and crystal field strength are strongly related to the environment of the lanthanide metal ion. The coordination geometry of the Dy atom in the complexes **4**, **8**, **15** and **16** is close to the triangular dodecahedron (TDD-8). Comparison of distortion of the [DyO_8_] polyhedron from the TDD-8 shape with thermal barrier energy did not reveal correlations between *S*_Q_(TDD-8) and *U*_eff_ values (Table 4 and Table S13 and Figure 12). Considering that the coordination geometry of the Dy atom as a square antiprism (SAPR-8) shows that decreasing S_Q_(SAPR-8) values correlates with increasing energy barriers, the highest energy barrier is found for **4** with R^F^ = CF_3_ with the lowest *S*_Q_(SAPR-8) value. An increase in fluorinated substituent size (R^F^ ≥ C_2_F_5_) in **8** and **15** or change of Li substituent to H_2_O in **16** leads to distortion of the [DyO_8_] polyhedron from the SAPR-8 shape, and thereby decreases the thermal barrier *U*_eff_. Therefore, both variations of Li and diketonate substituents affect the [DyO_8_] local symmetry as a result of modified crystal packing, and the greater correspondence to the ideal TDD-8 shape of the Dy^III^ polyhedron does not promote SIM behavior of the complex, whereas the square antiprismatic coordination environment is more favorable for higher thermal barrier *U*_eff_. 

## 3. Experimental

### 3.1. Materials and Methods

All reactions were carried out in air. Lanthanide salts TbCl_3_·6H_2_O (99.99%), DyCl_3_·4H_2_O (99.99%) were obtained from Alfa Aesar (Lancashire, UK), EuCl_3_·6H_2_O (99.99%) and Gd(NO_3_)_3_·5H_2_O (99.9%), which were obtained from Merck and used without further purification. 

The lithium β-diketonates (L^1^, L^2^, L^3^, L^4^) were synthesized according to the previously reported procedures [77,78,79,80].

IR diffuse-reflectance spectra were recorded with a Perkin-Elmer Spectrum One FTIR instrument in the range 400–4000 cm^−1^. Fluorescence and phosphorescence spectra were recorded in the solid state on a Varian Cary Eclipse fluorescence spectrophotometer with mutually perpendicular beams. The emission lifetimes ($\tau^{obs}$) and quantum yields ($Q_{Ln}^{L}$) have been measured using FS5 Edinburgh Instruments spectrofluorometer at room temperature with absolute error ±2%; excitation was performed through a ligand, and the absolute method in the integration sphere was used. Elemental analysis was performed using a Perkin Elmer (Waltham, MA, USA) PE 2400 Series II analyzer.

The single-crystal X-ray diffraction studies of **5**, **6**, **9**–**14** were carried out on a Bruker D8 Venture diffractometer (Mo-K_α_, λ = 0.71073 Å, graphite monochromator, ω/2θ-scanning technique). The intensity data for the single crystals of **8** and **15** were collected by the standard procedure on SMART APEX II CCD (Bruker AXS, Billerica, MA, USA) automated diffractometers (Mo Kα radiation, graphite monochromator, *T* = 240 K) [81]. Semiempirical absorption correction for **5**, **6**, **9**–**14** was applied for all compounds [82]. The structures were solved by direct methods and refined by the full-matrix least squares in the anisotropic approximation for non-hydrogen atoms. The calculations were carried out by the SHELX-2014/2018 program package [83] using Olex2 1.2/1.3 [84]. The hydrogen atoms of the ligands were positioned geometrically and refined using the riding model. Some restrictions were applied when solving the structure (DFIX (for **5**, **6**, **9**–**14**), DELU (for **5**, **6**, **10**, **12**, **13**), ISOR (for **5**, **6**, **10**, **12**, **13**), SADI (for **6**, **9**–**11**, **13**), EADP (for **9**, **11**, **14**), DANG (for **6**, **9**–**14**)). The crystal structure of **5** was solved taking into account the disordering of the methyl and methoxy groups at the C6 (here and below, their population (determined from the Fourier synthesis, except for **8**) corresponds to 0.66(2):0.34(2)), C24 (0.771(19):0.229(19)) and C33 (0.76(2):0.24(2)) atoms). The crystal structure of **6** was solved by taking into account the disordering of the methyl and methoxy groups at the C15 (0.68(3):0.32(3)) and C24 (0.76(2):0.24(2)) atoms, and 1,1-dimethoxyethyl group at the C3 atom (0.62(3):0.38(3)). The crystal structure of **8** was solved by taking into account the disordering of fluorine atoms in the CF_2_- and CF_3_-groups at the C3C, C3D, C6A, C6C, and C6D atoms (0.5:0.5). The crystal structure of **9** was solved by taking into account the disordering of the 1,1-dimethoxyethyl group at the C3 atom (0.847(6):0.153(6)), the 1-methoxyethyl groups at the C13 (0.874(6):0.126(6)) and C33 (0.561(4):0.439(4)) atoms, and part of the diketonate group (O13, C31 and C32 atoms) with the perfluoropenthyl group (0.657(2):0.343(2)). The crystal structure of **10** was solved by taking into account the disordering of the methyl and methoxy groups at the C7 (0.874(7):0.126(7)), C17 (0.932(9):0.068(9)) and C27 (0.874(7):0.126(7)) atoms, the 1-methoxyethyl group at the C32 atom (0.545(10):0.455(10)), and the perfluoropenthyl group at the C30 atom (0.591(4):0.409(4)). The crystal structure of **11** was solved by taking into account the disordering of the 1,1-dimethoxyethyl group at the C3 atom (0.828(9):0.172(9)), the 1-methoxyethyl groups at the C13 (0.859(9):0.141(9)) and C33 (0.515(5):0.485(5)) atoms, and part of the diketonate group (O13, C31 and C32 atoms) with the perfluoropenthyl group (0.518(3):0.482(3)). The crystal structure of **12** was solved by taking into account the disordering of the methyl and methoxy groups at the C8 (0.864(8):0.136(8)), C19 (0.834(9):0.166(9)) and C41 (0.945(8):0.055(8)) atoms. The crystal structure of **13** was solved by taking into account the disordering of the methyl and methoxy groups at the C8 (0.849(7):0.151(7)) and C41 (0.827(8):0.173(8)) atoms, and two fluorine atoms in the CF_3_-group at the C7 atom (0.41(2):0.59(2)). The crystal structure of **14** was solved by taking into account the disordering of the 1-methoxyethyl group at the C34 atom (0.583(8):0.417(8)), the methyl group at the O15 atom (0.583(8):0.417(8)), and fluorine atom in the CF_2_-group at the C20 atom (0.583(8):0.417(8)). 

Powder X-ray diffraction data were collected using a Bruker D8 Advance diffractometer (Bruker, Billerica, MA, USA) (CuKα, λ = 1.54 Å, Ni-filter, LYNXEYE detector, geometry reflection).

The magnetic susceptibility of the polycrystalline samples was measured with a Quantum Design MPMSXL SQUID magnetometer in the temperature range 2–300 K in the magnetic field of 5 kOe. Diamagnetic corrections were made using the Pascal constants. The effective magnetic moment was calculated as *µ_eff_*(T) = [(3k/NAµB2)χT]1/2 ≈ (8χT)^1/2^, where k is Boltzman constant, N_A_—Avogadro’s number and µ_B_—Bohr magneton. Check of the field dependence ac-susceptibility in the range 200–2000 Oe revealed that optimal dc magnetic field is 1 kOe. Therefore, frequency-dependent ac susceptibilities for complexes **8**, **15** and **16** were measured under 1 kOe dc field at various temperatures.

### 3.2. Synthesis of the Compounds ***1***–***16*** (General Method)

For a solution of LiL (1 mmol) in 15 mL of methanol (for **1**–**15**) or ethanol (for **16**), the Ln^III^ salt (0.25 mmol) was added and the mixture was stirred at room temperature for 1 h. The resulting solution was slowly evaporated, and solids were washed with water and cold methanol. The polycrystalline products were recrystallized from the corresponding alcohol (MeOH or EtOH) and filtered off through Celite^®^ 545 to afford a clear solution. Its slow evaporation at 5–10 °C gave colorless or slightly colored crystals suitable for single-crystal X-ray diffraction structure analysis. 

[LiEu(L^2^)_4_(MeOH)] (**5**). Yield 45%. Found: C, 33.97; H, 3.23. Calc. for C_37_H_44_EuF_20_LiO_17_: C, 34.19; H, 3.41.

[LiGd(L^2^)_4_(MeOH)] (**6**). Yield 53%. Found: C, 33.88; H, 3.21. Calc. for C_37_H_44_F_20_GdLiO_17_: C, 34.06; H, 3.40.

[LiDy(L^2^)_4_(MeOH)] (**8**). Yield 58%. Found: C, 33.73; H, 3.15. Calc. for C_37_H_44_DyF_20_LiO_17_: C, 33.92; H, 3.39.

[LiEu(L^3^)_4_(MeOH)] (**9**). Yield 51%. Found: C, 32.63; H, 2.75. Calc. for C_41_H_44_EuF_28_LiO_17_: C, 32.84; H, 2.96.

[LiGd(L^3^)_4_(MeOH)] (**10**). Yield 49%. Found: C, 32.56; H, 3.05. Calc. for C_41_H_44_F_28_GdLiO_17_: C, 32.72; H, 2.95.

[LiTb(L^3^)_4_(MeOH)] (**11**). Yield 61%. Found: C, 32.49; H, 2.78. Calc. for C_41_H_44_TbF_28_LiO_17_: C, 32.69; H, 2.94.

[LiEu(L^3^)_4_(MeOH)] (**12**). Yield 57%. Found: C, 31.64; H, 2.39. Calc. for C_45_H_44_EuF_36_LiO_17_: C, 31.80; H, 2.61.

[LiGd(L^4^)_4_(MeOH)] (**13**). Yield 50%. Found: C, 31.46; H, 3.21. Calc. for C_45_H_44_F_36_GdLiO_17_: C, 31.70; H, 3.35.

[LiTb(L^4^)_4_(MeOH)] (**14**). Yield 54%. Found: C, 31.45; H, 2.44. Calc. for C_45_H_44_TbF_36_LiO_17_: C, 31.67; H, 2.60.

[LiDy(L^4^)_4_(MeOH)] (**15**). Yield 44%. Found: C, 31.47; H, 2.35. Calc. for C_45_H_44_DyF_36_LiO_17_: C, 31.60; H, 2.59.

## 4. Conclusions

Based on acetal-containing β-diketonates of variable perfluoroalkyl chain length, the discrete heterometallic [Ln-Li] complexes were synthesized. The fluoroalkyl group affects both the crystal packing structure and the distortion of the coordination geometry in complexes. Increasing the number of fluorine atoms in the β-diketonates has no significant impact on the triplet-state energy level of the ligand. By increasing the length of fluoroalkyl substituent in the ligand, the distortion of the coordination geometry [LnO_8_] from ideal symmetry in [Ln-Li] complexes is irregular and depends on the nature of the Ln^III^ ion. In the series of Eu^III^ and Dy^III^ complexes, PLQY increases when the coordination polyhedron [LnO_8_] changes from TDD-8 to BTRP-8 geometry. However, the opposite correlation was observed in case of Tb^III^ complexes **7**, **14**: the closer the [TbO_8_] environment is to TDD-8 symmetry, the higher the PLQY (60–64%). It has been shown that decreasing the S_Q_(SAPR-8) value of coordination polyhedron [DyO_8_] correlates with increasing *U_eff_* values in Dy^III^ complex **4**.

## Figures and Tables

**Table 1:** A list of complexes **1**–**16** with CCDC numbers.

Compound	L^n^	R^F^	Ln	CCDC	Ref.
**1**	L^1^	CF_3_	Eu	1855391	[66]
**2**	L^1^	CF_3_	Gd	2031096	[68]
**3**	L^1^	CF_3_	Tb	1855392	[66]
**4**	L^1^	CF_3_	Dy	1855393	[66]
**5**	L^2^	C_2_F_5_	Eu	2258787	this work
**6**	L^2^	C_2_F_5_	Gd	2258788	this work
**7**	L^2^	C_2_F_5_	Tb	2011088	[70]
**8**	L^2^	C_2_F_5_	Dy	2255120	this work
**9**	L^3^	C_3_F_7_	Eu	2258789	this work
**10**	L^3^	C_3_F_7_	Gd	2258790	this work
**11**	L^3^	C_3_F_7_	Tb	2258791	this work
**12**	L^4^	C_4_F_9_	Eu	2258792	this work
**13**	L^4^	C_4_F_9_	Gd	2258793	this work
**14**	L^4^	C_4_F_9_	Tb	2258794	this work
**15**	L^4^	C_4_F_9_	Dy	2255119	this work
**16**	L^1^	CF_3_	Dy	1855396	[66]

**Table 2:** Continuous shape measures (CShM) calculations values for **Ln-Li** complexes using SHAPE.

	R^F^ = CF_3_	R^F^ = C_2_F_5_	R^F^ = C_3_F_7_	R^F^ = C_4_F_9_
[Eu**L**_3_)(Li**L**)(MeOH)]	1.315 (TDD-8), *D*_2d_ 1.983 (BTPR-8), *C*_2v_	1.055 (TDD-8), *D*_2d_	2.052/1.877 ^1^ (BTPR-8), *C*_2v_	1.932 (BTPR-8), *C*_2v_ 2.015 (TDD-8), *D*_2d_
[Gd**L**_3_)(Li**L**)(MeOH)]	1.253 (TDD-8), *D*_2d_ 1.941 (BTPR-8), *C*_2v_	1.003 (TDD-8), *D*_2d_	1.728 (BTPR-8), *C*_2v_	1.868 (BTPR-8), *C*_2v_ 1.963 (TDD-8), *D*_2d_
[Tb**L**_3_)(Li**L**)(MeOH)]	1.134 (TDD-8), *D*_2d_	0.986 (TDD-8), *D*_2d_	1.966/1.764 ^1^ (BTPR-8), *C*_2v_	0.947 (TDD-8), *D*_2d_
[Dy**L**_3_)(Li**L**)(MeOH)]	1.144 (TDD-8), *D*_2d_	0.894 (TDD-8), *D*_2d_	-	0.911 (TDD-8), *D*_2d_
[Dy**L**_3_)(Li**L**)(H_2_O)]	1.777 (TDD-8), *D*_2d_ 1.990 (BTPR-8), *C*_2v_	-	-	-

^1^ Two S_Q_(BTRP-8) values are due to the disordering of oxygen atoms in [LnO_8_].

**Table 3:** The radiative ($A_{rad}$) and non-radiative ($A_{nrad}$) decay rates, lifetimes ($\tau^{obs}$), intrinsic ($Q_{Ln}^{Ln}$) and overall ($Q_{Ln}^{L}$) quantum yields and sensitization effeciency (${ŋ}_{sens}$) of the [Ln-Li] complexes.

Complex	Ln	R^F^	$\tau^{obs}$, *μs*	$A_{rad}$, _s_^−1^	$A_{nrad}$, _s_^−1^	$Q_{Ln}^{L}$ **(%)**	$Q_{Ln}^{Ln}$ (%)	${ŋ}_{sens}$ (%)
**1**	Eu	CF_3_	690	836	613	53 ± 1	58	91
**5**		C_2_F_5_	701	969	458	39 ± 0.8	68	57
**9**		C_3_F_7_	655	949	578	52 ± 1	62	84
**12**		C_4_F_9_	703	900	522	55 ± 1	63	87
**3**	Tb	CF_3_	608	-	-	35 ± 0.7	-	-
**7**		C_2_F_5_	523	-	-	64 ± 1.3	-	-
**11**		C_3_F_7_	405	-	-	47 ± 0.9	-	-
**14**		C_4_F_9_	541	-	-	60 ± 1.2	-	-
**4**	Dy	CF_3_	6	-	-	1 ± 0.02	-	-
**16**		CF_3_	8	-	-	3 ± 0.06	-	-
**8**		C_2_F_5_	4	-	-	1 ± 0.02	-	-
**15**		C_4_F_9_	4	-	-	1 ± 0.02	-	-

**Table 4:** Best fit values of relaxation parameter values for Orbach and Raman processes.

Complex	L^n^	R^F^	*U_eff_*, cm^−1^	*τ*_0_, 10^−6^ s	*C*, s^−1^K^−*n*^	*n*	τ at 4 K, ms
**8**	**L^2^**	C_2_F_5_	21 ± 1	11 ± 1	0.029 ± 0.008	5.5 ± 0.2	10.4 ± 0.1
**15**	**L^4^**	C_4_F_9_	22 ± 1	9 ± 3	0.09 ± 0.01	5.23 ± 0.07	5.4 ± 0.1
**16**	**L^1^**	CF_3_	19 ± 1	14 ± 1	0.0011 ± 0.0001	7 (fixed)	12.6 ± 0.3

## Data Availability

Supplementary crystallographic data for the compounds synthesized are given in CCDC numbers 2258787 (**5**), 2258788 (**6**), 2255120 (**8**), 2258789 (**9**), 2258790 (**10**), 2258791 (**11**), 2258792 (**12**), 2258793 (**13**), 2258794 (**14**), 2255119 (**15**); These data can be obtained free of charge from The Cambridge Crystallographic Data Centre via www.ccdc.cam.ac.uk/data_request/cif, accessed on 1 June 2023.

## Supplementary Materials

The following supporting information can be downloaded at: https://www.mdpi.com/article/10.3390/ijms24119778/s1.
